# Renal hypoperfusion and impaired endothelium-dependent vasodilation in an animal model of VILI: the role of the peroxynitrite-PARP pathway

**DOI:** 10.1186/cc8932

**Published:** 2010-03-26

**Authors:** Rosanna Vaschetto, Jan W Kuiper, René JP Musters, Etto C Eringa, Francesco Della Corte, Kanneganti Murthy, AB Johan Groeneveld, Frans B Plötz

**Affiliations:** 1 Department of Clinical and Experimental Medicine, University of Eastern Piedmont "Amedeo Avogadro", Corso Mazzini 18, 28100, Novara, Italy; 2 Department of Pediatric Intensive Care, Vrije Universiteit Medical Center, De Boelelaan 1117, 1081 HV, Amsterdam, The Netherlands; 3 Department of Intensive Care, Vrije Universiteit Medical Center, De Boelelaan 1117, 1081 HV, Amsterdam, The Netherlands; 4 Institute for Cardiovascular Research, Vrije Universiteit Medical Center, De Boelelaan 1117, 1081 HV, Amsterdam, The Netherlands; 5 Department of Physiology, Vrije Universiteit Medical Center, De Boelelaan 1117, 1081 HV, Amsterdam, The Netherlands; 6 Inotek Pharmaceuticals Corporation, 33 Hayden Avenue, 0242, Lexington, MA, USA

## Abstract

**Introduction:**

Mechanical ventilation (MV) can injure the lungs and contribute to an overwhelming inflammatory response, leading to acute renal failure (ARF). We previously showed that poly(adenosine diphosphate-ribose) polymerase (PARP) is involved in the development of ventilator-induced lung injury (VILI) and the related ARF, but the mechanisms underneath remain unclear. In the current study we therefore tested the hypothesis that renal blood flow and endothelial, functional and tissue changes in the kidney of rats with lipopolysaccharide (LPS)-induced lung injury aggravated by MV, is caused, in part, by activation of PARP by peroxynitrite.

**Methods:**

Anesthetized Sprague Dawley rats (n = 31), were subjected to intratracheal instillation of lipopolysaccharide at 10 mg/kg followed by 210 min of mechanical ventilation at either low tidal volume (6 mL/kg) with 5 cm H_2_O positive end-expiratory pressure or high tidal volume (19 mL/kg) with zero positive end-expiratory pressure in the presence or absence of a peroxynitrite decomposition catalyst, WW85 or a PARP inhibitor, PJ-34. During the experiment, hemodynamics and blood gas variables were monitored. At time (t) t = 0 and t = 180 min, renal blood flow was measured. Blood and urine were collected for creatinine clearance measurement. Arcuate renal arteries were isolated for vasoreactivity experiment and kidneys snap frozen for staining.

**Results:**

High tidal volume ventilation resulted in lung injury, hypotension, renal hypoperfusion and impaired renal endothelium-dependent vasodilation, associated with renal dysfunction and tissue changes (leukocyte accumulation and increased expression of neutrophil gelatinase-associated lipocalin). Both WW85 and PJ-34 treatments attenuated lung injury, preserved blood pressure, attenuated renal endothelial dysfunction and maintained renal blood flow. In multivariable analysis, renal blood flow improvement was, independently from each other, associated with both maintained blood pressure and endothelium-dependent vasodilation by drug treatment. Finally, drug treatment improved renal function and reduced tissue changes.

**Conclusions:**

The peroxynitrite-induced PARP activation is involved in renal hypoperfusion, impaired endothelium-dependent vasodilation and resultant dysfunction, and injury, in a model of lung injury.

## Introduction

Mechanical ventilation (MV) remains the cornerstone of treatment in patients with acute lung injury (ALI) [1]. Animal and clinical studies show that MV can further injure the lungs, causing ventilator-induced lung injury (VILI) and can contribute to a systemic inflammatory response and development of multiple organ dysfunction syndrome [2-5]. The kidney is one of the organs most commonly involved [6,7]. There are few experimental studies addressing the role of MV in the development of acute renal failure (ARF) [2,5,8-10]. Multiple mechanisms could link VILI with ARF but specific contributions are difficult to ascertain [11]. There is increasing evidence that renal endothelial dysfunction plays a significant role in the development of ARF [12-14]. With injury, the endothelial cell loses its ability to modulate vasomotor and inflammatory responses [12-14].

In previous experimental studies, we described a fall in renal blood flow during injurious MV of normal lungs [10], and benefits of poly(ADP-ribose) polymerase (PARP) inhibitor given as pre-treatment on renal function and tissue integrity in lipopolysaccharide (LPS)-induced lung injury with superimposed MV [5], but their relation remains unclear. Indeed, the PARP pathway is activated both in VILI and ARF [5,15-18].

Oxygen and nitrogen-derived reactive species, such as peroxynitrite, induce oxidative DNA damage and consequent activation of the nuclear enzyme PARP. PARP overactivation is detrimental by depleting cellular ATP stores, resulting in cell dysfunction and death [19,20]. Thereby, activation of the pathway leads to endothelial dysfunction, as described in a wide variety of models [21-23]. Although PJ-34 is a pharmacological inhibitor of PARP independent on the activating stimuli [5,16], WW85 is a novel metalloporphyrinic peroxynitrite decomposition catalyst, releasing of NO_3_. The compound thus blocks peroxynitrite and thereby reduces PARP activation [24-26].

Peroxynitrite formation and PARP activation in lungs of animals with VILI have been demonstrated before [5,16,27]. To our knowledge, renal mechanisms involved in VILI-associated ARF and in particular related to the activation of PARP by peroxynitrite have not been studied before. Our current study extends previous observations [5] by further exploring the route of PARP inhibition involved in renal hemodynamic during LPS-induced lung injury aggravated by MV. We tested the hypothesis that renal blood flow and endothelial, functional and tissue changes in the kidney of rats with LPS-induced lung injury aggravated by MV, is caused, in part, by activation of PARP by peroxynitrite. We demonstrated that inhibition of PARP activation by peroxynitrite attenuates VILI and renal hypoperfusion and dysfunction, by maintaining endothelium-dependent vasodilation and decreasing inflammation and tissue injury.

## Materials and methods

### Animal preparation

**Figure 1:**
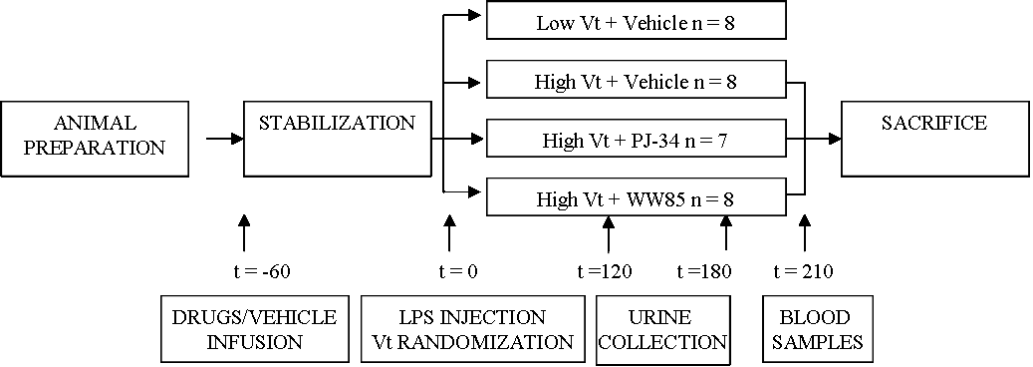
**Timeline of the protocol**. Animals were anesthetized, a tracheotomy was performed and animals were connected to a ventilator and ventilated in volume-controlled mode at 6 ml/kg, 5 cmH_2_O positive end-expiratory pressure. Arterial and venous catheters were inserted. One hour before lipopolysaccharide intratracheal injection, vehicle control or WW85 or PJ-34 were infused. At t = 0 minute, mechanical ventilation setting was changed according to the randomization and renal blood flow was measured. From t = 120 minute to t = 180 minutes urine was collected and blood samples were taken. At time t = 180 minutes renal blood flow was measured with different fluorescence microspheres. At the end of the experiment, at t = 210 minutes, blood samples were taken, animals were sacrificed, organs were harvested and arcuate renal arteries were isolated. Vt, tidal volume.

The experimental setup is shown in Figure 1. Animals were treated according to national guidelines and with permission of the Institutional Animal Care and Use Committee (Amsterdam, The Netherlands). A total of 31 male Sprague Dawley rats (Harlan CPB, Zeist, The Netherlands) with a mean weight of 310 ± 10 g, were anesthetized with a bolus of 60 mg/kg pentobarbital sodium (Nembutal; CEVA Santa Animale BV, Maassluis, The Netherlands) given intraperitoneally (ip) and 70 mg/kg ketamine (Alfasan, Woerden, The Netherlands) intramuscularly. Anesthesia was maintained with pentobarbital at 15 mg/kg every 30 minutes through an ip catheter and ketamine intravenously (iv) 20 mg/kg/h via tail vein; muscle relaxation was achieved by iv administration of pancuronium bromide 0.6 mg/kg/h. Rats were placed in the supine position on a heating pad, maintaining body temperature at 37°C. A tracheostomy was performed and a cannula (14 gauge) was inserted into the trachea. The right jugular vein, right carotid artery, and left femoral artery were cannulated with polyethylene tubing. The right jugular vein catheter and the left femoral artery catheter were connected to pressure transducers. Central venous pressure, mean arterial pressure (MAP) and heart rate were continuously monitored during the experiment. An acetone-stripped pulmonary artery catheter leaving only the thermistor was placed in the thoracic aorta via the right femoral artery. The bladder was catheterized for urine sampling using a transabdominal approach. Blood gas analysis was performed using a pH/blood-gas analyzer (ABL 50; Radiometer, Copenhagen, Denmark).

### Experimental protocol

PJ-34 was purchased from Alexis Biochemicals, Lausen, Switzerland. WW85 was kindly provided by Inotek Pharmaceuticals Corporation, Beverly, MA, USA. The rats were initially ventilated at a tidal volume (Vt) of 6 mL/kg and positive end-expiratory pressure (PEEP) of 5 cmH_2_O (AVEA Ventilator, Viasys Healthcare, Yorba Linda, CA, USA). Rats were randomly allocated into four groups: Vt 6 ml/kg and PEEP 5 cmH_2_O or Vt 19 ml/kg, no PEEP treated with either vehicle, PJ-34 or WW85 (Figure 1). For the control group, we adopted a relatively low Vt (6 ml/kg) plus PEEP following current clinical practice to minimize VILI. A second group was ventilated with high Vt (19 ml/kg) and zero PEEP, which is known to induce VILI [28,29] but has been used in the past years to maintain adequate oxygenation and normocapnia [30].

After a one-hour period, during which the animal was prepared and invasive monitoring was placed, drugs or vehicle bolus infusion was started: PJ-34 was administered iv as a loading dose of 10 mg/kg over 30 minutes, WW85 was administered 0.8 mg/kg ip. After one hour, a baseline arterial blood gas was measured to confirm similar gas-exchange conditions in all rats. LPS (055:B5, Sigma-Aldrich, St Louis, MO, USA) at 10 mg/kg in 0.5 ml normal saline was administered by using an intratracheal aerosolizer (PennCentury Inc, Philadelphia, PA, USA). Five minutes later, a recruitment manoeuvre was performed by increasing PEEP level to 25 cmH_2_O for five breaths, followed by 10 minutes of stabilization under the ventilator settings described above. Thereafter ventilation setting was changed according to the randomization and continued for 3.5 hours. PJ-34 was administered iv as a continuous infusion at 2 mg/kg/h for the remainder of the experiments [31]. Partial pressure of arterial carbon dioxide (PaCO_2_) was maintained at 40 ± 5 mmHg by adjusting the respiratory rate. The inspiration to expiration ratio was set to 1:2 and the fraction of inspired oxygen (FiO_2_) was kept at 0.45 for the whole experiment. Only in the case of a partial pressure of arterial oxygen (PaO_2_)/fraction of inspired oxygen (FiO_2_) inferior to 150 was FiO_2 _increased to 0.60. Administration of fluids was kept to a minimum, and did not differ between the groups. Approximately 1.5 mL/h normal saline per animal was infused to replace blood samples and flush intravascular catheters. Upon completion of the MV, the animals were sacrificed with an overdose of anesthetic. Right kidneys were snap frozen and stored at -80°C for histological examination. Left kidneys were immediately processed to isolate renal arcuate arteries. Plasma and urine were stored at -80°C until assayed. Lungs and heart were removed *en-bloc*. The right middle lobe was used to estimate wet/dry weight ratio.

### Cardiac output and renal blood flow measurements

Cardiac output (CO) (Cardiac Output Computer 9520A, Edwards Lifesciences, Irvine, CA, USA) was obtained every 60 minutes using the thermodilution method; 200 μl of cold saline was injected via the right jugular vein catheter as described previously [32]. Renal blood flow was measured at the randomization and at the end of the experiments using FluoSpheres polystyrene microspheres (15 μm scarlet fluorescent (645/680) and 15 μm blue-green fluorescent (430/465), Molecular Probes Europe, Leiden, The Netherlands). Renal blood flow in the left and right kidneys was calculated using a reference blood sample as previously described in detail, [33] and is expressed as the mean renal blood flow. The blood flow from the left and right triceps muscles was used to assess microsphere distribution.

### Renal functional parameters

Urine samples were collected from the 120^th ^to the 180^th ^minute after randomization, after emptying the urine tube. Arterial blood sample was collected at the 180^th ^minute. The samples were analyzed for sodium, creatinine, and urea (Modular Analytics, Roche Diagnostics, Mannheim, Germany). In rats with preserved urinary production, creatinine clearance was calculated using the formula U_Cr _× V/P_Cr_. In this formula U_Cr _represents the urine creatinine concentration (mg/mL), V is the urine flow (mL/min) and P_Cr _is the plasma creatinine concentration.

### Vasoreactivity experiments

To elucidate the contribution of endothelial damage via the peroxynitrite-PARP pathway, renal arcuate arteries were isolated (n= 6/group) and mounted in a pressure myograph. The mean arterial diameter was not different among groups (320 ± 20 μm). Diameter reponses of arteries to various stimuli under 37°C were measured as previously described [34]. 3-(N-morpholino)propanesulfonic (MOPS) buffer was used (in mM: 145 NaCl, 5 KCl, 2 CaCl, 1 MgSO_4_, 1 NaH_2_PO_4_, 3 MOPS, 2 pyruvate, 10 glucose, and 0.02 EDTA, pH 7.4) to fill the arteriole and pressure column. The organ chamber was filled with Krebs buffer (in mM: 110 NaCl, 5 KCl, 2.5 CaCl, 1 MgSO_4_, 1 KH_2_PO_4_, 10 glucose, 0.02 EDTA, and 24 NaHCO_3_, gassed with 95% air 5% CO_2_, pH 7.4). Vascular smooth muscle contractile function was studied by performing a cumulative concentration-response curve to determine norepinephrine sensitivity.

As a measure of norepinephrine sensitivity, we determined the -log EC50 value; this is the norepinephrine concentration at which the artery is constricted by 50%. This norepinephrine constriction level was used to test the endothelium-dependent vasodilatation with acetylcholine. The arteries were exposed to concentrations of acetylcholine ranging from 10^-8.5 ^to 10^-5.5 ^mol/L. Diameter changes were recorded until a steady state was reached. Dilations are expressed as a percentage of basal diameter (dia) = [(dia_acetylcholine _- dia_norepinephrine_)/(dia_basal _- dia_norepinephrine_)] × 100.

### Kidney staining

Kidney cryosections (5 μm; duplicate of n = 4/group) were fixed in formaldehyde 4% (Sigma-Aldrich, St. Louis, MO, USA). Common leukocyte antigen CD45 (AbD Serotec, Düsseldorf, Germany) or neutrophil gelatinase-associated lipocalin (NGAL) (Santa Cruz Biotechnology, Inc., Santa Cruz, CA, USA) antibody was incubated 1: 25 in PBS overnight at 4°C and washed three times in PBS with 0.05% Tween (PBST, Sigma-Aldrich, St. Louis, MO, USA) for five minutes. Thereafter, the sections were incubated for one hour with Alexa Fluor 488 conjugated anti-mouse or anti-rabbit depending on the primary antibody (Molecular Probes Europe, Leiden, The Netherlands) 1:100 in PBS. As a negative control a section with no primary antibody was used. After staining, sections were rinsed three times in PBST and incubated with rhodamine-conjugated wheat germ agglutinin (WGA, Molecular Probes Europe, Leiden, The Netherlands) for 20 minutes. Finally after five minutes washes in PBST, the sections were mounted on standard glass slide using Vectashield™ hard set mounting medium (Vector Laboratories, Burlingame, CA, USA) containing DAPI nuclear staining. Kidney sections were examined with Zeiss Axiovert 200 M Marianas™ inverted microscope (Carl Zeiss, Jena, Germany). Microscopy was performed with a 10 × air lens. The microscope, camera, and data were controlled by SlideBook™ software (SlideBook™ version 4.0.8.1 (Intelligent Imaging Innovations, Denver, CO, USA). SlideBook software was used to determine the mean fluorescence intensity.

### Statistics

Results are reported as median ± interquartile range. Data were analyzed in non-parametric tests by using Prism Graphpad 4.0 software package (Prism, San Diego, CA, USA). Comparison among groups was performed using Kruskal-Wallis test. When an overall *P *< 0.05, a Dunn's multiple-comparison *post hoc *analysis was conducted. A *P *value less than 0.05 was considered statistically significant. To assess the relative contribution of MAP, CO, acetylcholine responses and treatment, in the prediction of renal blood flow by these factors, we performed generalized estimating equations, taking repeated measures in the same animals into account. A *P *value less than 0.05 was considered significant.

## Results

### Lung injury by LPS and MV

**Figure 2:**
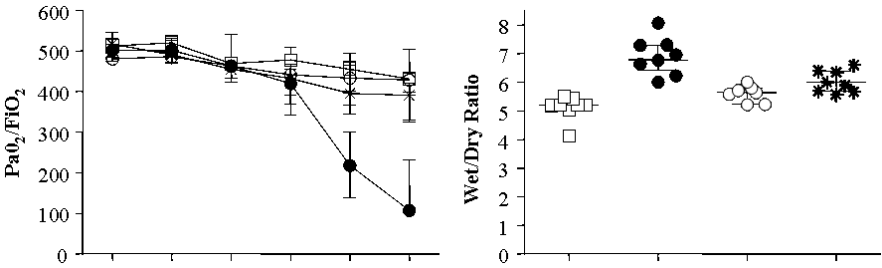
**Effects of WW85 or PJ-34 on respiratory mechanic and lung edema**. n = 8/group in low tidal volume (Vt)+Vehicle, high Vt+Vehicle, high Vt+WW85, n = 7/group in high Vt+PJ-34. **(a)**. Partial pressure of arterial oxygen (PaO_2_)/fraction of inspired oxygen (FiO_2_) ratio over time. * *P *< 0.05 high Vt+Vehicle vs. others.**(b) **Lung wet to dry weight ratio. * *P *< 0.05 high Vt+Vehicle vs. all. Values represent median (interquartile range).

The experimental setup is shown in Figure 1. Mean values of PaO_2_/FiO_2 _ratio were similar in all animals until the 120^th ^minute of MV when the PaO_2_/FiO_2 _started decreasing in the high Vt+Vehicle group compared with the other groups (Figure 2a). There were no differences in the levels of PaCO_2 _and pH among groups (data not shown). The lung wet/dry ratio was higher in the high Vt+Vehicle than in the low Vt+Vehicle group, and the treatment with the peroxynitrite decomposition catalyst or PARP inhibitor attenuated lung edema (Figure 2b).

### Hemodynamics variables

**Figure 3:**
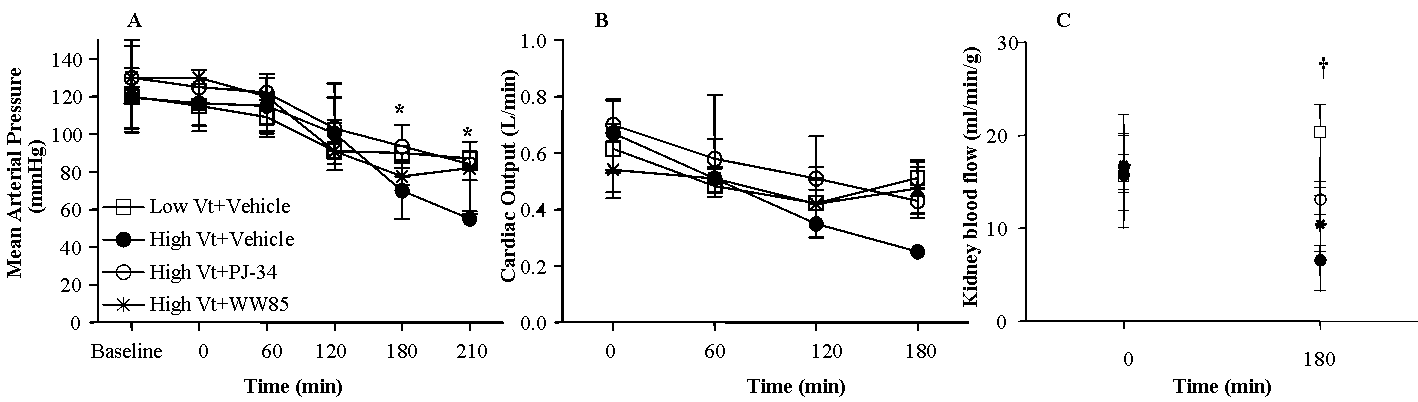
**Effects of WW85 or PJ-34 on hemodynamics**. Rats received lipopolysaccharide (10 mg/kg) intratracheally at time 0, followed by mechanical ventilation. n = 8/group in low tidal volume (Vt)+Vehicle, high Vt+Vehicle, high Vt+WW85, n = 7/group in high Vt+PJ-34. **(a) **Mean arterial pressures. * *P *< 0.05 high Vt+Vehicle vs. all at time 180 and 210 minutes. **(b) **Cardiac output over time. **(c) **Renal blood flow at time t = 0 and t = 180 minutes. † *P *< 0.05 high Vt+Vehicle vs. low Vt+Vehicle and high Vt+Vehicle vs. high Vt+PJ-34. n = 5/group. Values represent median (interquartile range).

MAP at baseline was similar among groups. After 180 minutes, MAP decreased in the high Vt+Vehicle group compared with the low Vt+Vehicle group (Figure 3a). WW85 or PJ-34 both attenuated the drop in MAP in the high Vt groups. There were no differences in CO among groups (Figure 3b).

Renal blood flow did not differ among groups at t = 0. After 180 minutes, the renal blood flow was 6.6 ml/min/g tissue (3.3 to 8.2 ml/min/g tissue) in the high Vt+Vehicle group, which was approximately 68% lower (*P *< 0.05) compared with the low Vt+Vehicle group, 20.4 ml/min/g tissue (13.5 to 23.2 ml/min/g tissue). WW85 or PJ-34 treatments preserved renal blood flow at 10.6 (7.6 to 14.3 ml/min/g tissue) and 13.2 ml/min/g tissue (11.5 to 15.1 ml/min/g tissue), respectively (Figure 3c).

### Endothelium-dependent vasodilation of renal arteries *ex vivo*

**Figure 4:**
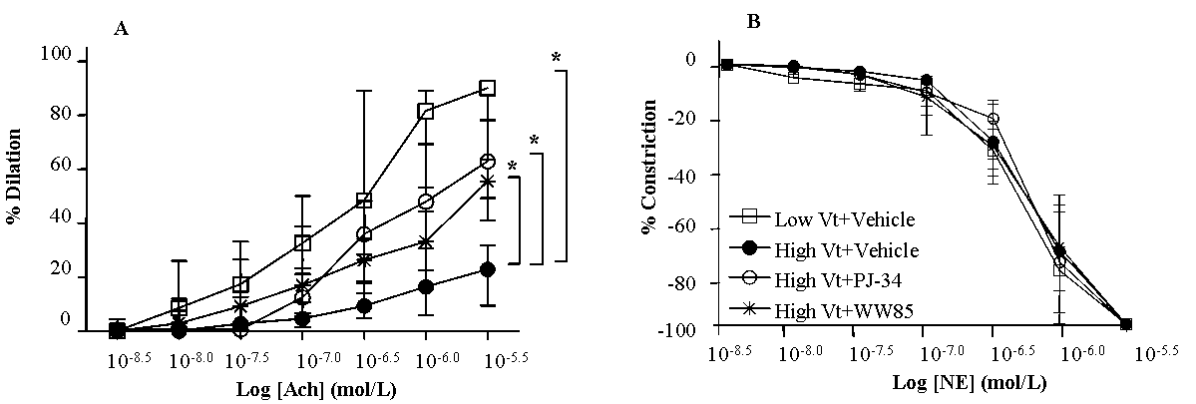
**Concentration-response curves**. **(a) **Concentration-response curves for norepinephrine (NE) of isolated renal arcuate arterioles. n = 5/group. **(b) **Concentration-response curves for acetylcholine (Ach) of isolated renal arcuate arterioles. ACh responses were tested in a pressure myograph after 50% preconstriction with NE. n = 5/group. * *P *< 0.05 high Vt+Vehicle vs. all. Vt, tidal volume. Values represent median (interquartile range).

Endothelium-dependent vasodilation of renal arcuate arteries, as indicated by the acetylcholine response, was decreased in high Vt+Vehicle group compared to low Vt+Vehicle control group. The acetylcholine response was conserved in high Vt groups treated with WW85 or PJ-34 (Figure 4a). The norephineprine-induced vasoconstriction response did not differ among the groups (Figure 4b).

### Renal function

**Figure 5:**
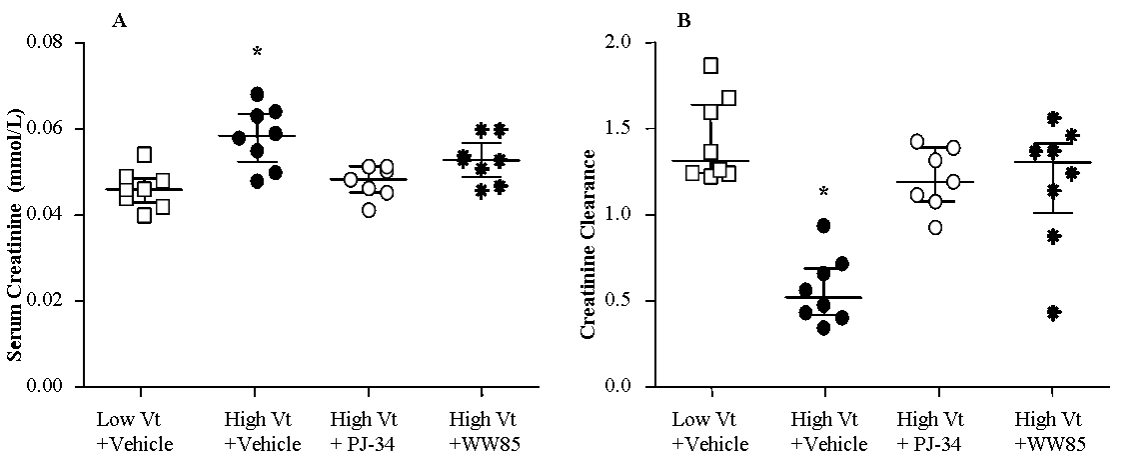
**Renal function**. **(a) **Serum creatinine at t = 180 minutes. **(b) **Creatinine clearance was measured over t = 120 minutes to t = 180 minutes. Creatinine clearance = U_Cr _× V/P_Cr_, where U_Cr _represents the creatinine concentration in urine (mmol/L), V the urine flow (mL/min), and P_Cr _the creatinine concentration in plasma (mmol/L). n = 8/group in low tidal volume (Vt)+Vehicle, high Vt+Vehicle, high Vt+WW85, n = 7/group in high Vt + PJ-34. * *P *< 0.05 high Vt+Vehicle vs. all. Values represent median (interquartile range).

The serum creatinine increased in the high Vt+Vehicle compared with the low Vt+Vehicle (Figure 5a) and creatinine clearance decreased in the former compared with the latter (Figure 5b). Treatment with either WW85 or PJ-34 preserved the increase in serum creatinine and prevented the fall in creatinine clearance. Blood urea nitrogen and fractional excretion of sodium did not differ among groups (data not shown).

### Leukocyte accumulation and NGAL expression in renal tissue

**Figure 6:**
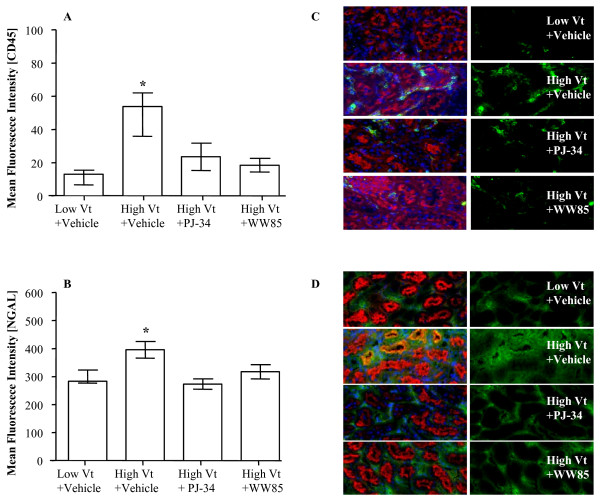
**Quantitative analysis**. **(a) **CD45. **(b) **Neutrophil gelatinase-associated lipocalin (NGAL) staining. Duplicate of n = 4/group. * *P *< 0.05 high tidal volume (Vt)+Vehicle vs. all. Values represent median (interquartile range). Representative kidney sections (10× air lens). Red staining: wheat germ agglutinin; blue staining: nuclei; green staining: **(c) **CD45, **(d) **NGAL.

The quantitative analysis of fluorescence intensity of CD45, a leukocyte marker, shows that the total amount of CD45-positive cells, mainly localized in corticomedullary area, was increased in the high Vt+Vehicle as compared with the low Vt+Vehicle group. Treatment with WW85 or PJ-34, in the former, decreased leukocyte infiltration to a level comparable with that of the latter (Figure 6a). We found an increase in NGAL tubular expression in rats ventilated with high Vt+Vehicle compared with those ventilated with low Vt+Vehicle, which was blunted by the administration of WW85 or PJ-34 (Figure 6b). Histological sections did not reveal other signs of injury (data not shown), as often happens in these short-time double hit models [35,36].

### Multivariable analyses

Although MAP (and not CO) was a major contributor to predict renal blood flow in time (*P *= 0.003), incorporating acetylcholine responses revealed that acetylcholine responses independently (*P *= -0.006) contributed to prediction of renal blood flow, together with MAP and drug treatments (*P *< 0.001). Conversely, the acetylcholine response was, independently of MAP (*P *= 0.006), predicted by drug treatment (*P *< 0.001).

## Discussion

Our current study suggests that hypoperfusion, impaired endothelial vasodilation, and associated functional and tissue changes in the kidney of rats with LPS-induced lung injury aggravated by MV, are caused, in part, by activation of PARP by peroxynitrite.

In our model, we instilled LPS intratracheally to induce pulmonary inflammation, followed by a high Vt and zero PEEP as injurious MV as conducted before [5]. VILI was characterized by diffuse alveolar lung injury as shown by a fall in PaO_2_/FiO_2 _ratio and lung edema compared with low Vt ventilation plus PEEP. However, severe hypoxemia (PaO_2 _<40 mm Hg) never occurred and PaCO_2 _was kept in a normal range in order to avoid alterations in renal blood flow due to changes in gas exchange [11]. Furthermore, to avoid the hemodynamic consequences of increased thoracic pressures, we applied the same mean airway pressures in the ventilated groups. As a result, the CO was similar among the groups.

Peroxynitrite formation and PARP activation in lungs of animals with VILI have been demonstrated before [5,16,27] and our current study extends previous observations [5] by further exploring the route of PARP inhibition involved in renal hemodynamics during LPS-induced lung injury aggravated by MV. Only a few studies explored vascular dysfunction in VILI, in particular norepinephrine- and acetylcholine-induced impaired aortic vascular responses [37-40] and impaired acetylcholine-induced pulmonary microvascular responses [40]. In these animal models, very large Vt of 35 ml/kg were applied to healthy rats to induce VILI during one hour of MV, leading to hypotension and microvascular hyperpermeability. The mechanism involved in these vascular alterations seems to be the consequence of intracellular reactive oxygen species and peroxynitrite formation, reversed, *in vitro*, by free-radical scavengers [37]. Other studies using lower Vt to injure the lung (15 to 17 ml/kg) in both healthy [10] or pre-injured animals [2,5,8,9] failed to show a decrease in blood pressure.

To our knowledge our study is the first to address renal microvascular responses during VILI. The renal changes evoked in our model were characterized by renal hypoperfusion, impaired endothelium-dependent vasodilation and associated dysfunction and tissue changes.

These observations may warrant a discussion of potential cause-effect relations in a complex model of inter-organ crosstalk. The model was characterized by global systemic vasodilation, in which release of soluble factors may be involved, and this may have directly contributed to the fall in renal blood flow. The data suggest that impaired endothelium-dependent vasodilation also contributed to this fall. However, we cannot definitively ascertain whether the beneficial effect of the two drugs on endothelium-dependent vasodilation and renal blood flow was caused by a direct protective effect on renal endothelium rather than by an anti-inflammatory effect preserving renal blood flow independent of endothelial changes. Our multivariable analysis suggests a direct protective effect on renal endothelium was the cause. It remains therefore unclear how the endothelium-dependent vasodilation is impaired. One possibility is that factors derived from the lung spill over into the systemic circulation, reach the kidney and evoke endothelial changes, but factors generated in the kidney and sensitive to the peroxynitrite-PARP pathway may also play a role [41,42]. Together with positive effects on MAP, acetylcholine response and, thereby, renal blood flow, drug treatment to inhibit the peroxynitrite-PARP pathway also inhibited inflammatory and tissue changes in the kidneys that may have contributed to the observed fall in renal function judged by creatinine clearance. Leukocyte accumulation and NGAL expression, detected predominantly in proximal tubule cells in response to tubular epithelial damage, are commonly observed in models of renal injury and dysfunction [43,44]. Indeed, in our study, we can not exclude also an endothelial expression of NGAL.

Few limitations of the study should be taken into account. First, we studied the peroxynitrite-PARP pathway in an experimental rat model of VILI, often employed in this contest [5,8,45-48]. Further research in humans is needed before these results can be translated to human medicine [49]. Second, taking into account the possible gender differences with respect to PARP activation found in animal models of stroke and LPS-induced inflammation, the results discussed previously might be applicable only to males [50-52]. Finally, although unlikely according to the literature, we can not exclude that WW85 or PJ-34 affect microcirculatory hemodynamics with other mechanisms other than through catalysation of peroxynitrite decomposition and PARP inhibition, respectively.

## Conclusions

In conclusion, our data suggest that inhibition of PARP activation by peroxynitrite attenuates VILI and renal hypoperfusion and dysfunction, by maintaining endothelium-dependent vasodilation and decreasing inflammation and tissue injury, in the rat kidney during LPS-induced lung injury aggravated by MV.

## Key messages

• VILI complicating ALI remains associated with high mortality rates and with the development of multiple organ failure. The kidney is one of the first organs to fail. The mechanisms that link MV with kidney failure are only speculated.

• The PARP pathway is activated in different models of ALI and ARF.

• In an animal model of lung injury, the pharmacological inhibition of peroxynitrite or PARP attenuated lung injury, preserved blood pressure, attenuated renal endothelial dysfunction and maintained renal blood flow, improving kidney function and reducing tissue changes.

• Renal blood flow improvement was, independently from each other, associated with both maintained blood pressure and endothelium-dependent vasodilation by drug treatment.

## Abbreviations

ALI: acute lung injury; ARF: acute renal failure; CO: cardiac output; FiO_2_: fraction of inspired oxygen; ip: intraperitoneally; iv: intravenously; LPS: lipopolysaccharide; MAP: mean arterial pressure; MV: mechanical ventilation; NGAL: neutrophil gelatinase-associated lipocalin; PaCO_2_: partial pressure of carbon dioxide; PaO_2_: partial pressure of oxygen; PARP: poly(adenosine diphosphate-ribose) polymerase; PBS: phosphate-buffered saline; PBST: phosphate-buffered saline and Tween; PEEP: positive end-expiratory pressure; Vt: tidal volume; VILI: ventilator-induced lung injury.

## Competing interests

Kanneganti Murthy has stock options and employment with Inotek Pharmaceuticals Corporation. All other authors declare that they have no competing interests.

## Authors' contributions

RV, FDC, JWK, ABJG and FBP have made substantial contributions to conception and design, acquisition of data, analysis and interpretation of data. RJPM and ECE have made substantial contributions to acquisition and analysis of data. RV, FDC, ABJG, KM and FBP have been involved in drafting the manuscript and revising it critically for important intellectual content. All authors read and approved the final manuscript.
